# Patterns of Integrative Korean Medicine Practice for Anxiety Disorders: A Survey among Korean Medicine Doctors (KMDs) in Korea

**DOI:** 10.1155/2020/3140764

**Published:** 2020-10-05

**Authors:** Sang-Il Seo, Sung-Youl Choi, Eun-Jung Kim, Byung-Soo Koo, Hyo Won-Jung, Gun-Hee Lee, Geun-Woo Kim

**Affiliations:** ^1^Department of Neuropsychiatry, Dong-Guk University Bundang Korean Medicine Hospital, 268, Buljeong-ro, Bundang-gu, Seongnam-si, Gyeonggi-do 13601, Republic of Korea; ^2^Department of Neuropsychiatry, College of Korean Medicine, Gachon University, 1342, Seongnam-daero, Sujeong-gu, Seongnam-si, Gyeonggi-do 13120, Republic of Korea; ^3^Department of Acupuncture &Moxibustion, College of Korean Medicine, Dong-Guk University, 32, Dongguk-ro, Ilsandong-gu, Goyang-si, Gyeonggi-do 10326, Republic of Korea; ^4^Department of Neuropsychiatry, College of Korean Medicine, Dong-Guk University, 32, Dongguk-ro, Ilsandong-gu, Goyang-si, Gyeonggi-do 10326, Republic of Korea; ^5^Department of Herbology, College of Korean Medicine, Dong-Guk University, 123, Dongdae-ro, Gyeongju-si, Gyeongsangbuk-do 38066, Republic of Korea; ^6^Sogang Business School, Sogang University, 35, Baekbeom-ro, Mapo-gu, Seoul 04107, Republic of Korea

## Abstract

Anxiety disorder is known as the most common disease among psychiatric disorders. However, many studies have not been conducted in the Korean medicine area. This study explores the current state of anxiety disorder treatments of Korean medicine through a survey research. The survey for Korean medicine doctors (KMDs) on Korean medicine (KM) diagnosis and treatments for anxiety disorder was conducted online from December 21, 2016, to December 29, 2016. The results were divided into two groups, KMDs and Korean medicine neuropsychiatric specialists (KMNPS), and comparatively analyzed. Self-evaluation and counseling were the most common in both diagnostic methods and evaluation of treatment effects, and KMNPS tended to make extensive use of objective indicators. There was no difference in the rate of psychiatric medication use among the patients between KMD and KMNPS. The main reason for patients wanting KM treatment was the tapering cessation of psychiatric medications. The most common treatments were acupuncture, herbal medicine, and moxibustion, in addition to dry cupping in KMD and psychotherapy in KMNPS. The most important factor for treatment was herbal medicine treatment, followed by rapport formation in KMD and patient's temperament in KMNPS. Opinions on various items were presented as treatment barriers, and KMNPS tended to think more importantly about the patient's family problems. For the items to be additionally trained in the future, KMD chose the diagnostic tools and KMNPS chose psychotherapies. This study is the first study to analyze the clinical patterns for anxiety disorder in KMDs. KMD and KMNPS showed similar patterns in the perception, diagnosis, and treatment of anxiety disorders, but KMNPS tended to use objective indicators and psychotherapy more actively. Further clinical studies for the development of clinical guidelines should be additionally required.

## 1. Introduction

Anxiety disorder is the most common disease among psychiatric disorders. According to the WHO report, the proportion of the global population with anxiety disorders in 2015 is estimated to be 3.6% [1], and the total estimated number of people living with anxiety disorders in the world is 264 million. This total for 2015 reflects a 14.9% increase since 2005 [2]. A systematic analysis showed that the pooled period prevalence was 6.7% (6.0–7.6%) from 122 surveys with a lifetime prevalence of 12.9% (11.3–14.7%) from 70 surveys [3]. It is also shown that the lifetime prevalence of anxiety disorders in Korea has been reported as 9.3% (male 6.7% and female 11.7%) with the annual prevalence rate at 5.7% (male 3.5% and female 7.5%). That is almost two times higher than depression, at lifetime prevalence of 5.4% (male 3.3% and female 7.2%), with an annual prevalence rate of 3.1% (male 1.8% and female 4.3%) according to the Epidemiological Survey of Mental Disorders. In addition, the lifetime prevalence of all mental illnesses decreased from 29.9% in 2001 to 26.6% in 2016, but the lifetime prevalence of “anxiety disorder” has increased to 6.9% in 2006, 8.7% in 2011, and 9.3% in 2016 [4]. However, the lifetime use rate of mental health services for patients with anxiety disorders is still low at 25.1% in 2011 and 27.3% in 2016 [5].

Korean medicine doctor (KMD) is a Korean medical practitioner who provides diagnosis, treatment, and prescription and necessary medical consultations to maintain the desired level of health [6]. A Korean medicine specialist is a person who is certificated in oriental medicine and has acquired professional qualifications through one year of internship with three years of residence training after 2000. Currently, specialists are trained in 8 subject areas (Internal Korean Medicine, Acupuncture and Moxibustion Medicine, KM Obstetrics and Gynecology, KM Pediatrics, KM Rehabilitation, KM Neuropsychiatry, Sasang Constitutional Medicine, KM Ophthalmology, Otolaryngology and Dermatology). KM specialists accounted for about 9.1% of all KM doctors by 2013 [7]. Training subjects of Korean medicine neuropsychiatry specialist (KMNPS) include psychophysiology, psychopathology, psychopharmacology, neurology with several other psychological-psychiatric theories and techniques, psychiatric examination, differential diagnosis, psychological testing, specialized KM therapies for neuropsychiatric disorders, and KM psychotherapies such as Qi-gong and meditation, Korean traditional psychological-psychiatric theories, personality theories, and techniques [8].

There are many types of psychiatric illnesses situated in KM institutions that are mostly related to physical symptoms. KM, which has a symptom-based approach to determine the treatment through the identification of specific etiology or specific symptoms rather than prioritizing the name of the disease [9], may have an advantage in anxiety disorders, which indicate the diversity of symptoms among psychiatric disorders. In fact, complementary and alternative medicine treatments including KM and Chinese medicine are used worldwide [10]. However, the standard of care has not been established due to subjective diagnosis and various treatment methods. Therefore, the pursuit of evidence-based care is a very important topic, and the demand for clinical practice guidelines (CPG) is increasing. In the Chinese version of the Evidence-Based Guidelines for Clinical Practice in Chinese Medicine, depressive disorders and insomnia CPG are mentioned, but none exists for anxiety disorders [11].

The purpose of this study is to analyze the KM treatment pattern of anxiety disorders and reflect the data in developing KM CPG for anxiety disorders. Also, the study is to investigate the possibility of clinical application of KM therapy tools based on the Diagnosis and Statistical Manual for Mental Disorders (DSM-IV TR and DSM-5) and International Classification of Diseases (ICD-10), after that the questionnaire consists of panic disorder (PD), posttraumatic stress disorder (PTSD), social anxiety disorder (SAD), and generalized anxiety disorder (GAD). Finally, we expect that this study will contribute to the standardization and efficiency improvement of KM treatment for anxiety disorders, enabling KMDs to provide the best medical care.

## 2. Materials and Methods

### 2.1. Study Design

This questionnaire was prepared for KMD and KMNPS to understand the perception and treatment of anxiety disorders. SIS searched for literature published within the last 10 years with the search formula [“anxiety disorder” or “generalized anxiety disorder” or “panic disorder” or “post-traumatic stress disorder” or “social phobia”] and [“professional” or “specialist” or “therap^∗^”] and “survey” in PubMed. Of the 219 search results, 12 relevant studies were selected and referenced in the production of the questionnaire [12–23]. Three KMNPS (SIS, SYC, and GWK) and one acupuncture and moxibustion medicine specialist (EJK), totaled four KMDs, participated in drafting. The questionnaire covers demographic characteristics, diagnosis method, treatment method, assessment method, and factors affecting the treatment of anxiety disorders. The draft was made after two conferences. One KMNPS (BSK) and one KMD (HWJ), who had not participated in the drafting, participated in revision. The revised version was finalized after consulting with KMNPS and statisticians who did not participate in the questionnaire production. The Supplementary Material (available here) shows the entire survey. This study was approved by the Institutional Review Board of Dongguk University Bundang Korean Hospital in Korea (DUBHIRB2017-0012).

### 2.2. Participants and Procedures

The questionnaire was distributed to 18,329 KMDs and 165 KMNPSs registered through the Association of Korean Medicine. We used “Surveymonkey,” an online survey platform, and the survey was conducted online from December 21, 2016, to December 29, 2016, and was sent twice via e-mail.

### 2.3. Statistical Analysis

All statistical analyses and data handling were performed using R version 3.4 (R Foundation for Statistical Computing, Vienna, Austria). The Chi-square test of independence was used to identify the statistical significance of characteristic differences between KMDs and KMNPSs groups. The characteristics include diagnostic methods, psychotropic drug use rates, and treatment methods, and treatment-related factors.

We divided the data into two groups, KMD and KMNPS, for comparative analysis, because it was assumed that there would be differences in treatment patterns between the two groups. Therefore, we tried to uncover the differences and use them as data to provide a complementary treatment process in the future.

## 3. Results

### 3.1. Demographic Characteristics

Statistical analysis was performed based on the survey data of 677 KMDs and 80 KMNPSs. The response rate was 3.69% for KMDs and 48.5% for KMNPSs. The average age of the KMD who participated in the questionnaire was 35.2% in their 40 s and 32.9% in their 30 s. In the KMNPS, the average age was in their 30 s with 63.8%, followed by those in their 40 s with 30.0%. The gender of the KMD was 80.1% in male, 19.9% in female, and of the KMNPS was 68.8% in male and 31.3% in female. The types of medical institutions worked were KM clinics 66%, KM hospitals 17.1% and long-term care hospitals 6.9% in KMDs, and KM clinics 45%, KM hospitals 28.8%, and long-term care hospitals 6.9% in KMNPSs, respectively. It is shown that clinical career did not differ by 12.97 ± 6.6 years for the KMD and average of 12.18 ± 8.64 years for the KMNPS. The degrees in KMD were 54.4% for bachelor, 26.4% for doctor, and 19.2% for masters. The degree in KMNPS was 51.3% for doctor, 28.8% for master, and 20.0% for bachelor. Demographic characteristics of respondents are shown in Table 1.

The average number of patients with anxiety disorder per month was 4.47 ± 4.56 in the KMD and 6.63 ± 5.72 in the KMNPS. The average number of first-visit patients with anxiety disorder was 8.95 ± 15.63 in the KMD and 14.31 ± 14.86 in the KMNPS. It is shown in Table 2.

### 3.2. Diagnostic Methods of Anxiety Disorder Patients

In the KMD, the diagnostic methods of patients with anxiety disorder were as follows: self-evaluation (87.7%) and counseling (72.8%), and they were as follows in the KMNPS: self-evaluation (86.3%), counseling (75.0%), DSM, ICD diagnostic criteria (56.3%), anxiety questionnaire (e.g., STAI, BAI, and HAM-A) (55.0%), instrumental examination (e.g., HRV, Neurofeedback, 33.3%). Anxiety questionnaire, semi- or structured interview, DSM·KCD diagnostic criteria, and instrumental examination (e.g., HRV and Neurofeedback) were used more significantly (*p* < 0.001) by KMNPS (Table 3).

### 3.3. Psychotropic Drug Use Rate in Anxiety Disorder Patients

The proportion of psychotropic medications in first-visit patients with anxiety disorder is almost the same as 48.31 ± 32.27% of the KMD and 47.88 ± 26.66% of the KMNPS (Table 4).

### 3.4. Treatments of Anxiety Disorder Patients

The reason why patients with anxiety disorder wants to be treated with oriental medicine is to stop or reduce the treatment of drugs (56.0% and 80.0%), improvement of physical symptoms (47.6% and 78.8%), improvement of psychological symptoms (46.4% and 61.3%), and the quality of life (46.4% and 43.8%) in both the KMD and the KMNPS. KMNPS answered significantly (*p* < 0.001) more in the improvement of physical symptoms and stop medication or decrease dosage (Table 5).

The most common treatment for anxiety disorder in both KMD and KMNPS is acupuncture (KMD 95.9% and KMNPS 98.8%) and herbal decoction (KMD 85.1% and KMNPS 91.3%). In the KMD, treatments are used in the order of dry cupping (71.5%), moxibustion (48.6%), pharmaco-acupuncture (24.8%). In the KMNPS, treatments are used in the order of psychotherapy (71.3%), moxibustion (60.0%), and NHIS exclusion herbal extracts (45.0%). Electroacupuncture, NHIS-excluded herbal extracts, wet cupping, aromatherapy, and physical therapy were significantly (*p* < 0.001) more used in KMNPS (Table 6).

KMD mainly used self-evaluation (93.1%) and counseling (75.9%) to evaluate anxiety disorder treatment. KMNPS used self-evaluation (96.2%), counseling (75.0%), tapering of antipsychotics (58.8%), and change in test scale (52.5%). KMNPS tended to use (*p* < 0.001) more change in test scale, tapering of psychotropic medicine to assess treatment effects (Table 7).

The most important factor in the treatment of anxiety disorder in KMD was herbal medicine treatment (43.1%) and rapport formation (39.9%), while in KMNPS, herbal medicine treatment (45.0%), patient's temperament (21.3%), and rapport formation (20.0%). Significant differences (*p* < 0.001) were found in the rapport formation with high response to KMD and patient's temperament with high response to KMNPS (Table 8).

The factors that interfere with the treatment of patients with anxiety disorders are patient symptom characteristics (49.9%), patient lifestyle (44.6%), patient family problem (43.9%), and patient personality characteristics (39.6%) in KMD. In the case of KMNPS, the factors are patient family problem (68.8%), patient personality characteristics (52.5%), patient symptom characteristics (46.2%), and patient lifestyle and slow treatment effect (37.5%). There was a significant difference (*p* < 0.001) in the patient family problems, which was highly answered by KMNPS (Table 9).

KMD chose the use of diagnostic tools the most (79.6%) as an area requiring additional education in the treatment of anxiety disorder and was significantly higher than KMNPS (*p* < 0.001). On the other hand, KMNPS answered psychotherapy (62.5%) as the most important additional education. Results are shown in Table 10.

## 4. Discussion

Before discussing the survey results, the KMD and KMNPS survey response rates were very different, 3.69% and 48.5%, respectively. Since the subject of the survey is anxiety disorders, one of the most frequent diseases in the psychiatric field, KMNPS majoring in psychiatry seems to have higher interest and response rates of the questionnaire than KMD.

In the demographic context, KMNPS accounted for the highest percentage in the 30 s because the KMNPS began to produce in 2000 and have had a shorter history. In the case of KMNPS, there were a relatively large number of workers working at the KM Hospital which has specialized departments. The relatively large number of doctor and master degree holder in the specialist are seen as an effort to have medical expertise (Table 1).

The assessment is as follows. As a diagnostic/evaluation tool used with anxiety disorders, both KMD and KMNPS account for a high proportion of self-evaluation of patients and information acquisition through counseling. Unlike KMD, KMNPS is more likely to use objective indicators such as DSM/ICD diagnostic criteria, questionnaires, and physical tests (Table 3). This can be seen as securing more patient information and providing objectivity of secured information.

The rate of psychotropic medication in patients with anxiety disorder was 48% in both groups, and the reason seems that the severity of the symptoms of anxiety disorder patients coming to KMD and KMNPS for treatment is considered to be the same (Table 4).

The reason for choosing KM treatment for anxiety disorders is the improvement of psychological and physical symptoms, along with cutting off or reducing the amount of psychotropic drugs. Although psychotropic medication is a universal treatment for the treatment of anxiety disorders, patients are often found to seek Korean medicine for reducing their reliance on psychotropic drugs or as an alternative treatment for adverse drug reactions (Table 5).

For the treatment tools used in the treatment of anxiety disorders, both KMD and KMNPS selected the treatment tools commonly used in Korean medicine such as acupuncture and herbal medicine. However, KMNPS relatively chose psychotherapy which requires professionalism in terms of its application (Table 6).

The most commonly used methods for evaluating the effectiveness of treatments are self-evaluation of patients and counseling in both KMD and KMNPS. In addition, KMNPS are more likely to use objective indicators such as psychotropic drug doses and changes in psychological scale measures; it is consistent with the characteristics that use objective indicators (Tables 3 and 7).

For the important factors in the treatment of anxiety disorders, both groups regarded herbal medicine treatment as the most important. While KMD emphasized herbal medicine treatment and rapport formation at an equal level, in the case of KMNPS, the treatment of herbal medicine is the most important, followed by the patient's temperament and the rapport formation (Table 8). KMNPS tend to prefer psychotherapy over KMD in the treatment of anxiety disorders (Table 6) and seem to focus on resources and making cooperative relationship with patients.

In questionnaire of the disturbances in the treatment of anxiety disorders, both groups reported that the various factors presented are meaningful. In KMNPS, the family problems, personality characteristics, accompanying physical illnesses, and slow treatment effects are considered as obstacles. In particular, the family problems and personality characteristics of patients are 68.8% and 52.5%, respectively, that is over half of them (Table 9). While current symptoms are the most important factor for KMD, KMNPS tends to place greater importance on family problems and personality traits in patients. Just as KMNPS consider the patient's temperament as an important factor in treatment, they can be thought to focus on factors that are more problematic than the symptoms themselves.

Finally, in the additional educational items that they think are necessary, KMD choose utilizing diagnostic tools the most and KMNPS choose psychotherapy the most (Table 10). This may mean that KMD is not accustomed to the Western neuropsychiatric diagnosis as compared to KMNPS. In the case of KMNPS, since they mainly used psychotherapy to treat patients with anxiety disorders (Table 6), so the need for new psychotherapeutic education appears to be for additional expertise.

There are several limitations in this survey. We surveyed the current state of KM treatment for anxiety disorders only in Korea. Therefore, China, Japan, and many other countries using traditional Asian medicine (TAM) have not been able to figure out how they perceive and treat anxiety disorders. In addition, this was an overview of how KMDs diagnose, treat, and evaluate anxiety disorders. It does not include specific and practical contents such as which acupuncture points, prescriptions, or psychotherapies are used frequently. Therefore, various treatment methods and patterns that are used empirically in the clinical field should be additionally considered in future survey for development of CPG.

This study is the first study on the current state of the medical practice of the TAM psychiatrist in Korea. The guideline currently under development will include the characteristics such as the rate of anxiety disorders patients in KM institution, their psychotropic drug use, and KM treatment tool type and utilization tendency. And this study illustrated the differences between KMD and KMNPS for anxiety disorders, so that it was possible to check what is needed to improve the ability for treating anxiety disorders. In the future, based on the results of this study, statistical studies of other diseases are also expected to be more diverse and in-depth analysis than before through comparison of specialists and general practitioners.

## 5. Conclusions

This study is the first study to analyze the current state of anxiety disorder treatment in KMDs. KMD and KMNPS showed similar patterns in the perception, diagnosis, and treatment of anxiety disorders, as both are studied and trained in the same oriental medicine. However, the KMNPS is more likely to use objective indicators and psychotherapies as they have been trained in neuropsychiatry for three years and has had experience in applying expertise to the treatment of anxiety disorders. Further clinical research is required to develop clinical guidelines utilized by KMD and KMNPS, respectively.

## Figures and Tables

**Table 1:** Demographic characteristics of respondents.

Classification	KMD (*N* = 677)	KMNPS (*N* = 80)
	*n* (%)/mean ± SD	*n* (%)/mean ± SD
Age		
Less than 30	111 (16.4)	0 (0.0)
30–39	223 (32.9)	51 (63.8)
40–49	238 (35.2)	24 (30.0)
50–59	5 (0.7)	5 (6.3)
More than 60	18 (2.7)	0 (0.0)
Sex		
Female	135 (19.9)	25 (31.3)
Male	542 (80.1)	55 (68.8)
Place of work		
Korean medical clinic	449 (66.3)	36 (45.0)
Korean medical hospital	116 (17.1)	23 (28.8)
Convalescence hospital	47 (6.9)	5 (6.3)
Others	65 (9.6)	16 (2.0)
Clinical experience		
Years	12.18 ± 8.64	12.97 ± 6.6
Education		
Bachelor	368 (54.4)	16 (20)
Master	130 (19.2)	23 (28.8)
Doctor	179 (26.4)	41 (51.3)

Values are expressed as *n* (%) for age, sex, place of work, and education and as mean (^∗^standard deviation) for clinical experience. KMD, Korean medicine doctor; KMNPS, Korean medicine neuropsychiatry specialist.

**Table 2:** Number of patients with anxiety disorders.

Classification	KMD(*N* = 677)	KMNPS (*N* = 80)
	*n* (%)	*n* (%)
Number of patients diagnosed with anxiety disorder per month		
0	178 (26.3)	12 (15.0)
1–9	461 (68.1)	52 (65.0)
10–19	29 (4.3)	13 (16.3)
20–29	5 (0.7)	3 (3.8)
More than 30	4 (0.6)	0 (0.0)
Mean (SD)	4.47 (4.56)	6.63 (5.72)
Number of patients complain of anxiety disorder as a symptom per month		
0	84 (12.4)	6 (7.5)
1–10	471 (69.6)	39 (48.8)
11–29	97 (14.3)	26 (32.5)
30–49	14 (2.1)	7 (8.8)
50–100	6 (0.9)	2 (2.5)
More than 100	5 (0.7)	0 (0.0)
Mean (SD)	8.95 (15.63)	14.31 (14.86)

Values are expressed as *n* (%) and mean (standard deviation). KMD, Korean medicine doctor; KMNPS, Korean medicine neuropsychiatry specialist.

**Table 3:** Diagnostic methods for patients with anxiety disorders.

Method	KMD (*N* = 677)	KMNPS (*N* = 80)	*p* value (*χ*^2^ test)
	*n* (%)	*n* (%)	
Self-evaluation	594 (87.7)	69 (86.3)	0.839
Anxiety questionnaire (e.g., STAI, BAI, HAM-A)	78 (11.5)	44 (55.0)	<0.001
Semi- or structured interview (e.g., SCID)	16 (2.4)	10 (12.5)	<0.001
DSM, KCD diagnostic criteria	92 (13.6)	45 (56.3)	<0.001
Counseling	493 (72.8)	60 (75.0)	0.778
Instrumental examination (e.g., HRV and Neurofeedback)	58 (8.6)	27 (33.8)	<0.001
Etc.	10 (1.5)	2 (2.5)	0.829

Values are expressed as *n* (%). *p* values are calculated by Chi-square test. KMD, Korean medicine doctor; KMNPS, Korean medicine neuropsychiatry specialist; STAI, state-trait anxiety inventory; BAI, Beck Anxiety Inventory; HAM-A, Hamilton Anxiety Rating Scale; SCID, structured clinical interview for DSM; KCD, Korean standard classification of diseases; HRV, heart rate variability.

**Table 4:** Percentage of patients taking psychiatric medications.

Classification	KMD (*N* = 677)	KMNPS (*N* = 80)
	*n* (%)	*n* (%)
0	39 (5.8)	3 (3.8)
1–10%	102 (15.1)	8 (10.0)
10–30%	91 (13.4)	9 (11.3)
30–60%	168 (24.8)	33 (41.3)
60–90%	175 (25.8)	22 (27.5)
Over than 90%	102 (15.1)	5 (6.3)
Mean (SD)	48.31 (32.27)	47.88 (26.66)

Values are expressed as *n* (%). KMD, Korean medicine doctor; KMNPS, Korean medicine neuropsychiatry specialist.

**Table 5:** The reason why patients want KM treatment.

Reason	KMD (*N* = 677)	KMNPS (*N* = 80)	*p* value (*χ*^2^ test)
	*n* (%)	*n* (%)	
Improving the quality of life	314 (46.4)	35 (43.8)	0.743
Improvement of psychological symptoms	314 (46.4)	49 (61.3)	0.016
Improvement of physical symptoms	322 (47.6)	63 (78.8)	<0.001
Stop medication or decrease dosage	379 (56.0)	64 (80.0)	<0.001
Dissatisfaction with modern medical treatment	292 (43.1)	39 (48.8)	0.402
Etc.	7 (1.0)	1 (1.3)	1

Values are expressed as *n* (%). *p* values are calculated by chi-square test. KMD, Korean medicine doctor; KMNPS, Korean medicine neuropsychiatry specialist.

**Table 6:** KM treatment methods used for anxiety disorders.

Treatment	KMD (*N* = 677)	KMNPS (*N* = 80)	*p* value (*χ*^2^ test)
	*n* (%)	*n* (%)	
Acupuncture	649 (95.9)	79 (98.8)	0.335
Electroacupuncture	132 (19.5)	32 (40.0)	<0.001
Ear acupuncture	79 (11.7)	12 (15.0)	0.494
Intradermal acupuncture	42 (6.2)	8 (10.0)	0.292
Warm acupuncture	31 (4.6)	2 (2.5)	0.568
Pharmacoacupuncture	168 (24.8)	26 (32.5)	0.176
Bee venom acupuncture	21 (3.1)	0 (0.0)	0.216
Chuna manipulation	79 (11.7)	14 (17.5)	0.186
Conduction exercise	16 (2.4)	7 (8.8)	0.005
56 NHIS inclusion herbal extracts	178 (26.3)	34 (42.5)	0.003
NHIS exclusion herbal extracts	170 (25.1)	36 (45.0)	<0.001
Chinese herbal decoctions	576 (85.1)	73 (91.3)	0.186
Wet cupping	147 (21.7)	33 (41.3)	<0.001
Dry cupping	484 (71.5)	28 (35.0)	0.017
Moxibustion	329 (48.6)	48 (60.0)	0.07
Aromatherapy	66 (9.7)	27 (33.8)	<0.001
Biofeedback	13 (1.9)	5 (6.3)	0.044
Psychotherapy	117 (17.3)	57 (71.3)	<0.001

Values are expressed as *n* (%). *p* values are calculated by Chi-square test. KMD, Korean medicine doctor; KMNPS, Korean medicine neuropsychiatry specialist; NHIS, National Health Insurance Service.

**Table 7:** Assessment of treatment effect of anxiety disorder patients.

Classification	KMD (*N* = 677)	KMNPS (*N* = 80)	*p* value (*χ*^2^ test)
	*n* (%)	*n* (%)	
Self-evaluation	63 (93.1)	77 (96.2)	0.396
Change in test scale	96 (14.2)	42 (52.5)	<0.001
Counseling	514 (75.9)	60 (75.0)	0.965
Tapering of psychotropic medicine	260 (38.4)	47 (58.8)	<0.001
Instrumental examination	62 (9.2)	13 (16.2)	0.07
Pulse diagnosis	156 (23.0)	22 (27.5)	0.453
Etc.	7 (1.0)	0 (0)	1

Values are expressed as *n* (%). *p* values are calculated by Chi-square test. KMD, Korean medicine doctor; KMNPS, Korean medicine neuropsychiatry specialist; NHIS, National Health Insurance Service.

**Table 8:** The most important factor in treating anxiety disorders.

Factor	KMD (*N* = 677)	KMNPS (*N* = 80)	*p* value (*χ*^2^ test)
	*n* (%)	*n* (%)	
Rapport formation	270 (39.9)	16 (20.0)	<0.001
Herbal medicine treatment	292 (43.1)	36 (45.0)	0.842
Acupuncture treatment	52 (7.7)	5 (6.3)	0.815
Patient's temperament	37 (5.5)	17 (21.3)	<0.001
Psychotherapy	14 (2.1)	6 (7.5)	0.013
Psychotropic medication	9 (1.3)	0 (0)	0.615

Values are expressed as *n* (%). *p* values are calculated by Chi-square test. KMD, Korean medicine doctor; KMNPS, Korean medicine neuropsychiatry specialist.

**Table 9:** Obstructive factors in the treatment of anxiety disorders.

Factor	KMD (*N* = 677)	KMNPS (*N* = 80)	*p* value (*χ*^2^ test)
	*n* (%)	*n* (%)	
Patient symptom characteristics	338 (49.9)	37 (46.2)	0.614
Patient personality characteristics	268 (39.6)	42 (52.5)	0.036
Patient lifestyle	302 (44.6)	30 (37.5)	0.275
Patient family problems	207 (43.9)	55 (68.8)	<0.001
Alcohol dependence	161 (23.8)	23 (28.7)	0.4
Accompanying physical disease	101 (14.9)	21 (26.2)	0.014
Slow treatment effect	177 (26.1)	30 (37.5)	0.043
Distrust of treatment	157 (23.2)	21 (26.2)	0.638
Inadequate expectations	175 (25.8)	27 (33.8)	0.168
Lack of motivation	181 (26.7)	26 (32.5)	0.336
Lack of rapport formation	161 (23.8)	23 (28.7)	0.4
Others	18 (2.7)	2 (2.5)	1

Values are expressed as *n* (%). *p* values are calculated by chi-square test. KMD, Korean medicine doctor; KMNPS, Korean medicine neuropsychiatry specialist.

**Table 10:** Additional training items needed for anxiety disorders treatment.

Training item	KMD (*N* = 677)	KMNPS (*N* = 80)	*p* value (*χ*^2^ test)
	*n* (%)	*n* (%)	
Use of diagnostic tool	539 (79.6)	34 (42.5)	<0.001
Use of instrumental examination	222 (32.8)	20 (25.0)	0.198
Use of psychotherapy	356 (49.6)	50 (62.5)	0.039
Details of psychiatry	253 (37.4)	37 (46.2)	0.155
Etc.	10 (1.5)	6 (7.5)	0.004

Values are expressed as *n* (%). *p* values are calculated by Chi-square test. KMD, Korean medicine doctor; KMNPS, Korean medicine neuropsychiatry specialist.

## Data Availability

The data used to support the findings of this study are available from the corresponding author upon request.

## Abbreviations

BAI: Beck Anxiety Inventory
CPG: Clinical practice guideline
DSM: Diagnostic and statistical manual of mental disorders
GAD: Generalized anxiety disorder
HAM-A: Hamilton Anxiety Rating Scale
HRV: Heart rate variability
ICD: International Classification of Diseases
KM: Korean medicine
KMD: Korean medicine doctor
KMNPS: Korean medicine neuropsychiatric specialist
NHIS: National Health Insurance Service
PD: Panic disorder
PTSD: Posttraumatic stress disorder
SAD: Social anxiety disorder
SCID: Structured Clinical Interview for DSM
STAI: State-trait anxiety inventory
TAM: Traditional Asian Medicine.

## Supplementary Materials

Supplementary Materials Survey for the Development of Standard Clinical Pathway Guideline for Korean Medicine with Anxiety Disorders.
