# Supplementary material for: Patterns of Integrative Korean Medicine Practice for Anxiety Disorders: A Survey among Korean Medicine Doctors (KMDs) in Korea
Source: Evid Based Complement Alternat Med. 2020 Oct 5;2020:3140764. doi: 10.1155/2020/3140764 (PMC7556402; doi:10.1155/2020/3140764)
Supplement: Supplementary Materials — Survey for the Development of Standard Clinical Pathway Guideline for Korean Medicine with Anxiety Disorders. [file 3140764.f1.pdf]

Survey for the Development of Standard Clinical Pathway Guideline for Korean Medicine with Anxiety Disorders

Hello.

This survey is conducted under the supervision of the Ministry of Health and Welfare and concerns the 'Development of Standard Clinical Pathway Guideline for Korean Medicine with Anxiety Disorder'.

Anxiety disorder is a disease that appears as various types of mental and physical symptoms in primary oriental medical institutions, and it requires standardized medical guidelines and enhanced security. Your answer to the survey will be used as important material in developing a standard Clinical Pathway Guideline for Korean medicine with anxiety disorders.

This research project is being done by the Industry-Academic Cooperation Foundation of Dongguk University, and we promise that the responses will not be used for purposes other than statistical purposes for research under Chapter 5, Section 33, of the Statistics Act.

Thank you for your response.

Research Director of Korean Medicine for Anxiety Disorders in Korea Clinical Care Guidelines

Geun-Woo Kim, Dongguk University Korean Medicine College

Co-researcher

Kyung-Ok Kim, Dongshin University Korean Medicine College

Hyung-Won Kang, Wonkwang University Korean Medicine College

Sung-Yeol Choi, Gachon University Korean Medicine College

Sang-Il Seo, Dongguk University Korean Medicine College

A. Doctor's Information

(1) How old are you?

- Younger than 30
- 30-39
- 40-49
- 50-59
- Older than 60

(2) What is your gender?

- Male
- Female

(3) Where do you work?

- Oriental Clinic
- Oriental Medicine Hospital
- Public health centers or branch offices (a public health doctor)
- Nursing hospital
- Taking a leave of absence.
- Other :

(4) How many years have you had clinical experience?

- Less than 5 years
- More than 5 years to less than 10 years
- More than 10 years to less than 20 years
- More than 20 years to less than 30 years
- More than 30 years

(5) What is your final educational background?

- Bachelor's
- Master's

- Doctorate's

B. Matters concerning anxiety disorders

The categories of anxiety disorders to be investigated in this research project are limited to 'panic disorder', 'post traumatic stress disorder', 'social anxiety disorder' and 'generalized anxiety disorder'.

Here are the questions about the status of patients with anxiety disorders on the first medical examination.

(1)-1 How many patients are diagnosed with anxiety disorder per month on the first medical examination?

- 0
- 1-9
- 10-19
- 20-29
- Over 30

(1)-2 How many patients a month have an anxiety disorder accompanied by symptoms?

- 0
- 1-10
- 11-29
- 30-49
- 50-100
- Over than 100

(3) What do you use as a diagnostic or evaluation tool for patients with anxiety disorder during the first medical examination?

Multiple responses are possible.

- Self-evaluation
- Anxiety questionnaire (ex. STAI, BAI, HAM-A)
- Semi or structured interview (ex. SCID)
- DSM, KCD diagnostic criteria
- Counselling

- Instrumental examination (e.g., HRV, Neurofeedback)

※ STAI : State-Trait Anxiety Inventory

※ BAI : Beck Anxiety Inventory

※ HAM-A : Hamilton Anxiety Scale

(4) What is the percentage of patients with anxiety disorder who are taking psychotropic drugs (including antidepressants, antidepressants, etc.)?

- 0
- 1-10%
- 10-30%
- 30-60%
- 60-90%
- Over than 90%

(5) Based on patient statements or experience, why would a patient with an anxiety disorder want Korean medical treatment?

Multiple responses are possible.

- To improve their quality of life
- To improve their psychological symptoms
- To improve their physical symptoms
- To stop their medication or decrease their dosage
- Dissatisfaction with modern medical treatment
- Other

(6) Select all Korean medical treatment methods that you usually use when visiting patients with anxiety disorder.

Multiple responses are possible.

- Acupuncture
- Electro-acupuncture
- Ear acupuncture

- Intradermal acupuncture
- Warm acupuncture
- Pharmaco-acupuncture
- Bee venom acupuncture
- Chuna manipulation
- Conduction exercise
- 56 NHIS inclusion herbal extracts
- NHIS exclusion herbal extracts
- Chinese herbal Decoctions
- Wet cupping
- Dry cupping
- Moxibustion
- Aromatherapy
- Biofeedback
- Psychotherapy

(7) How do you assess the effectiveness of treating anxiety disorders?

- Self-evaluations
- Changes in test scale (ex. STAI, BAI, HAM-A)
- Counselling
- Tapering of psychotropic medicine
- Instrumental examination
- Pulse diagnosis
- Etc.

※ STAI : State-Trait Anxiety Inventory

※ BAI : Beck Anxiety Inventory

※ HAM-A : Hamilton Anxiety Scale

(8) What was the most important factor when the progress in treating anxiety disorder was successful?

- Rapport formation

- Herbal Medicine treatment

- Acupuncture treatment

- Patient's temperament

- Psychotherapy

- Psychotropic medication

(9) What are obstructive factors in the treatment when treating an anxiety disorder?

(Multiple responses are available)

- Patient symptom characteristics

- Patient personality characteristics

- Patient lifestyle

- Patient family problems

- Alcohol dependence

- Accompanying physical disease

- Slow treatment effect

- Distrust of treatment

- Inadequate expectations

- Lack of motivation

- Lack of rapport formation

- Etc.

(10) What do you think you need additional training for treating anxiety disorders?

- Use of diagnostic tools

- Use of instrumental examination

- Use of Psychotherapy

- Details of Psychiatry

- Etc.
